# Identification of a *Sinorhizobium meliloti* YbgC-like thioesterase that contributes to the production of the infochemical 2-tridecanone

**DOI:** 10.1042/BCJ20253120

**Published:** 2025-09-22

**Authors:** Lydia M. Bernabéu-Roda, Geovanny Rivera-Hernández, Virginia Cuéllar, Rafael Núñez, Ángeles Moreno-Ocampo, Christian Sohlenkamp, Otto Geiger, María J. Soto, Isabel M. López-Lara

**Affiliations:** 1 Department of Biotechnology and Environmental Protection, Estación Experimental del Zaidín, CSIC, Granada, 18008, Spain; 2 Programa de Ecología Genómica, Centro de Ciencias Genómicas, Universidad Nacional Autónoma de México, Cuernavaca, Morelos, 62210, Mexico; 3 Scientific Instrumentation Service, Estación Experimental del Zaidín, CSIC, Granada, 18008, Spain

**Keywords:** methylketone biosynthesis, plant–bacteria interactions, *Sinorhizobium (Ensifer) meliloti*, Tol-Pal associated thioesterase, volatile compounds, YbgC

## Abstract

*Sinorhizobium meliloti* is a soil bacterium that can establish beneficial symbiosis with legume plants. The *fadD* gene encodes a long-chain fatty acyl-coenzyme A (CoA) synthetase. Inactivation of FadD in *S. meliloti* leads to a pleiotropic phenotype, including the overproduction of several volatile methylketones (MKs). One of them, 2-tridecanone (2-TDC), was found to act as an infochemical that affects important bacterial traits and hampers plant–bacteria interactions. Knowledge about bacterial genes involved in MK production is limited. In wild tomato species, MK synthesis requires intermediates of fatty acid biosynthesis and the activity of the methylketone synthase 2 (MKS2), a thioesterase belonging to the hotdog-fold family. In this study, we have identified SMc03960, a conserved hypothetical protein with homology to bacterial YbgC-like thioesterases, as an ortholog of MKS2 in *S. meliloti*. Heterologous expression of *smc03960* in *Escherichia coli* results in the formation of several MKs, including 2-TDC, and causes the accumulation of free fatty acids. Purified His-SMc03960 showed thioesterase activity for different acyl groups linked either to acyl carrier protein (ACP) or to CoA with preference for C14-long substrates. Moreover, formation of 2-TDC *in vitro* was achieved by using His-SMc03960 and 3-oxo-myristoyl-ACP. Although deletion of *smc03960* in the wild type or in the *fadD* mutant does not significantly alter the amount of MKs released by *S. meliloti*, overexpression of the gene results in increased production of 2-TDC in these two strains. Overall, our data demonstrate that SMc03960 is an acyl-ACP/acyl-CoA thioesterase with broad substrate specificity that contributes to 2-TDC formation.

## Introduction

The alpha-proteobacterium *Sinorhizobium* (syn. *Ensifer*) *meliloti* can engage in a beneficial nitrogen-fixing symbiosis with the legume host plant alfalfa. The establishment of this symbiosis is a complex process that requires a continuous molecular dialogue between the two partners [1]. During this interaction, the bacteria need to colonize the plant roots, a process that is influenced by several factors [2–4]. FadD is an acyl-coenzyme A (CoA) synthetase specific for long-chain fatty acids allowing them to enter β-oxidation [5]. Inactivation of the *fadD* gene in *S. meliloti* provokes an increase in surface motility, negatively affects biofilm development, and impairs alfalfa root colonization [6–9]. Studies aimed at identifying the molecular bases responsible for this pleiotropic phenotype revealed that *fadD* mutants of *S. meliloti* overproduce the methylketone 2-tridecanone (2-TDC) and that this compound acts as an infochemical that affects important bacterial traits and hampers plant–bacteria interactions [10,11]. Besides the participation of FadD in regulating 2-TDC levels in *S. meliloti*, enzymatic activities responsible for 2-TDC production in the alfalfa symbiont remain unknown.

Methylketones (MKs) are broadly distributed in nature, and their production has been reported in plants, fungi, bacteria, insects, and mammals [12]. Several MKs have been shown to exhibit relevant biological activities acting as natural insecticides in plants [13], affecting growth of plants and fungi [14], or increasing plant resistance to a/biotic stresses [11,15,16]. Moreover, these compounds are industrially valuable chemicals, which are used in the flavor and fragrance industry [17] and as promising biofuels [18,19]. Despite their extensive distribution and commercial interest, there are few reports about pathways and genes for the biosynthesis of MKs. 2-TDC was identified as a natural insecticide produced by wild tomato plants [20] and the first genes for 2-TDC biosynthesis were identified in these plants. Fridman et al. [21] observed increased expression of genes for the fatty acid biosynthetic pathway in glandular trichomes of wild tomato, indicating that MKs were derived from the intermediate of fatty acid biosynthesis 3-oxo-acyl-acyl carrier protein (ACP). Methylketone synthase 2 (MKS2) was identified as a thioesterase that hydrolyzes 3-oxo-acyl-ACPs to release 3-oxo-acids, whereas methylketone synthase 1 (MKS1) catalyzes the decarboxylation of these liberated 3-oxo-acids, generating the corresponding MK products, mainly 2-undecanone and 2-TDC [22]. It is well known that 3-oxo-acids are unstable and can undergo spontaneous decarboxylation, explaining that expression of only MKS2 in *E. coli* results in formation of MKs [22]. Like other members of the plastid-localized acyl-lipid thioesterase (ALT) class, MKS2 has a single hotdog-fold, whose signature is a five-stranded antiparallel β-sheet around an elongated α-helix [23]. Based on strong sequence similarity, tertiary structure, and conserved active sites and catalytic residues, MKS2 was classified into the type 9 family of hot dog-fold thioesterases (TE9) [24]. YbgC bacterial Tol-Pal-associated thioesterases are also members of the TE9 family. The Tol-Pal system is well conserved in Gram-negative bacteria and is required for maintaining cell envelope integrity, including outer membrane stabilization through its connection to peptidoglycan. Furthermore, the Tol-Pal system has a prominent role in completing bacterial cell division [25,26]. YbgC proteins from different bacteria have been shown to exhibit thioesterase activity on various acyl-CoA substrates [27–29]. However, the production of MKs associated with the activity of YbgC proteins has not been reported yet. Indeed, information about bacterial thioesterases with a dedicated role in MK production is limited. The volatile 2-heptanone produced by *Bacillus nematocida* B16 has the ability to attract nematodes. The thioesterase YneP was identified as a homolog of MKS2 in *B. nematocida,* and expression of *yneP* in *E. coli* led to the prominent formation of the MKs 2-pentanone, 2-heptanone, and 2-nonanone [30].

Here, we describe the involvement of the *S. meliloti* YbgC-like thioesterase SMc03960 in MK formation as well as in the release of free fatty acids and 3-hydroxy fatty acids. SMc03960 shows a broad substrate specificity being able to hydrolyze different acyl-CoAs and acyl-ACPs with a preference for 14-carbon substrates. In addition, we show that whereas the inactivation of *smc03960* in *S. meliloti* does not lead to a significant variation in MK release, its overexpression specifically results in increased production of 2-TDC.

## Results and discussion

### SMc03960, a sinorhizobial homolog of MKS2 from tomato, is a YbgC-like protein

**Figure 1:**
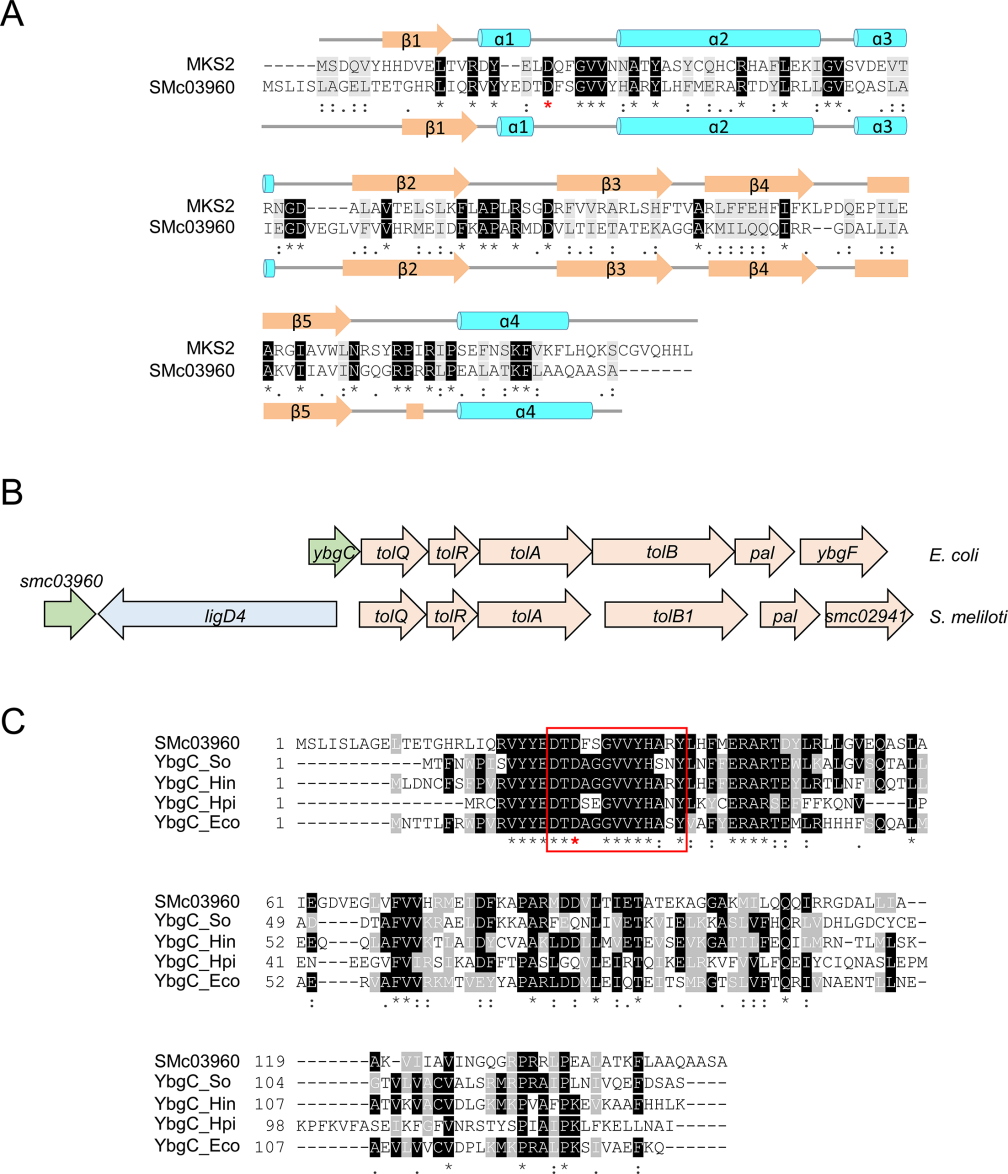
SMc03960 is homologous to MKS2 and to YbgC. (**A**) Amino acid sequence alignment of tomato methylketone synthase MKS2 (accession ADK38536.1, without transit peptide) and SMc03960 (accession WP_003527598.1). The predicted secondary structure elements of MKS2 and SMc03960 are shown above and below their corresponding amino acid sequences. (**B**) Genomic context of the genes for the Tol-Pal system in *E. coli* (above) and *S. melitoti* (below). (**C**) CLUSTAL Omega (1.2.4) multiple sequence alignment of SMc03960 with the YbgC proteins from *Helicobacter pylori* (YbgC_Hpi, WP_001203797.1), *Shewanella oneidensis* (YbgC_So, WP_011072691.1), *Haemophilus influenzae* (YbgC_Hin, WP_136440267.1) and from *E. coli* (YbgC_Eco, WP_001098384.1). The red square denotes the consensus sequence [DTD-X(2)-GVV-X-H-X(2)-Y] that defines the active site core of YbgC. In the alignments shown in (A) and (C) identical residues are highlighted with a black background and an asterisk, highly conserved residues are shown with a grey background and a colon, and partial matches with a full stop. The catalytic aspartate residue required for thioester bond cleavage in the bacterial thioesterase 4HBT [31] and mutated in SMc03960-D27A is indicated with a red asterisk.

Given that the inactivation of FadD abolishes the production of long-chain acyl-CoAs, accumulation of MKs in *S. meliloti fadD* mutants most likely derives from substrates (acyl-ACPs) and enzymatic reactions similar to those described for tomato plants [22]. In the case of 2-TDC production, the fatty acid biosynthesis intermediate 3-oxo-myristoyl-ACP might be hydrolyzed by an MKS2-like thioesterase resulting in the release of 3-oxo-myristic acid, which enzymatically or spontaneously decarboxylates to generate 2-TDC. To identify proteins that could play a role in 2-TDC synthesis in *S. meliloti*, the amino acid sequence of MKS2 without the chloroplast transit peptide was used as a query against the *S. meliloti* proteome database (https://iant.toulouse.inra.fr/bacteria/annotation/cgi/rhime.cgi) using the BLASTP algorithm. The search retrieved SMc03960 as a protein showing 25% identity within 80% coverage (E value = 1.9 x 10^-4^) with the tomato enzyme. An alignment with MUSCLE (3.8) of the sequence of both proteins is presented in Figure 1A. Furthermore, secondary structures for SMc03960 and MKS2 predicted using PSIPRED (v4.0) were found to be very similar (Figure 1A). SMc03960 is annotated as a conserved hypothetical 151 amino acid-long protein and it exhibits homology with Tol-Pal system-associated acyl-CoA thioesterases (referred to as YbgC) from different bacteria [27–29,32,33]. As in the case of *E. coli*, *ybgC* is frequently the first gene in the *tol-pal* gene cluster (Figure 1B). However, in *S. meliloti,* downstream of the *smc03960* gene and in the opposite orientation, a gene encoding a non-homologous end joining LigD protein (LigD4 [34,35]) is located, thereby interrupting the *tol-pal* gene cluster (Figure 1B). The five genes *tolQ, tolR, tolA, tolB1,* and *pal,* considered to constitute the core Tol-Pal system [25], are present in the *S. meliloti* genome (Figure 1B), and some of their components have been shown to be essential for cell growth [36]. SMc03960 shows 39% identity with *E. coli* YbgC, and it shares the consensus sequence [DTD-X(2)-GVV-X-H-X(2)-Y] that defines the core active site of YbgC proteins [37] Figure 1C). Overall, amino acid sequence similarity and the genomic context identify the MKS2-homolog SMc03960 to be the YbgC-like protein of *S. meliloti*.

### Expression of *smc03960* in *Escherichia coli* leads to the release of MKs and free fatty acids

**Figure 2:**
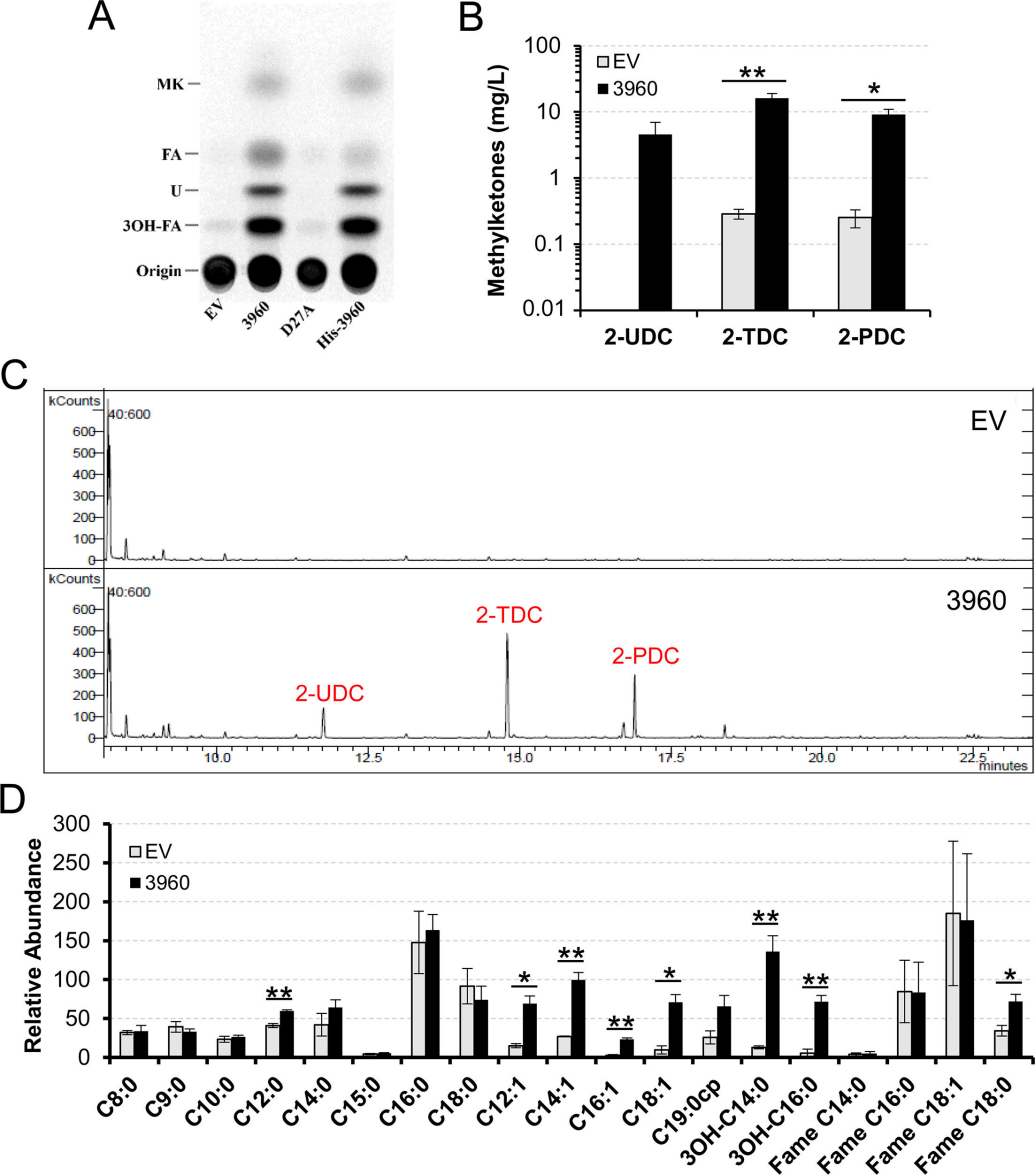
The expression of *smc03960* in *E. coli* causes methylketone and fatty acid accumulation in culture medium. (**A**) Radiolabeled-lipid profile analysis of spent culture media from *E. coli* BL21(DE3) × pLysS containing the empty vector pET17b (EV), the SMc03960-expressing construct pIML50 (3960), the construct pGRHD27A which expresses the site-directed mutant protein SMc03960_D27A (D27A), or the His-SMc03960-expressing construct pGRH01 (His-3960). The origin and comigration of the products with methylketones (MK), free fatty acids (FA), and 3-hydroxy fatty acids (3OH-FA) are indicated, U: unidentified compound. (**B**) Quantification of MKs present in the lipidic extracts from spent culture media of *E. coli* cells overexpressing (3960) or not (EV) *smc03960*. (**C**) Representative GC/MS chromatograms in the selected ion monitoring (SIM) mode for fragment ion at *m/z* 58 (specific for MK) of *E. coli* lipidic extracts obtained from spent medium of *E. coli* BL21(DE3) × pLysS containing the empty vector pET17b (EV) or the SMc03960-expressing construct pIML50 (3960). Absolute quantification was performed by the external standard method using calibration curves with authentic standards. (**D**) Relative abundance of free fatty acids and fatty acid methyl esters (FAME) present in lipidic extracts obtained from the spent medium of *E. coli* cultures overexpressing (3960) or not (EV) *smc03960*. Relative abundance of each compound was calculated using the FAME C13:0 as internal standard. In (B) and (D) data represent the average of three biological replicates, and error bars represent the standard error. Statistical significance relative to the control strain according to an ANOVA test is shown (*P<0.05, **P<0.005).

To determine the biochemical activity of SMc03960, the corresponding ORF was expressed in *E. coli* using the pET expression system. In the first instance, we analyzed by thin layer chromatography (TLC) compounds differentially formed four hours after induction with isopropyl β-D-1-thiogalactopyranoside (IPTG) in lipid extracts from cells and the spent medium of *E. coli* cultures. While there are no significant differences in lipid extracts obtained from cells, four spots were clearly increased in spent media from cultures expressing *smc03960* with respect to the *E. coli* strain carrying the empty vector pET17b (Supplementary Figure S1 and Figure 2A). By comparison with authentic standards, spots could be assigned to MKs, free fatty acids, and 3-hydroxy fatty acids, while the identity of one of the spots remained unknown (Supplementary Figure S1). In SMc03960, aspartic acid (D) 27 (Figure 1) is probably an essential residue required for thioesterase activity, as has been established by the findings that the replacement of the D27 counterpart by asparagine inactivates YbgC enzymes, as shown for *Hi*YbgC and *So*YbgC [27,29] or for the 4-hydroxybenzoyl-CoA thioesterase from *Pseudomonas* [31]. There is no significant difference in the lipid profile of spent media obtained from *E. coli* cells harboring the empty vector pET17b or a plasmid construct expressing the site-directed mutant SMc03960-D27A (Figure 2A). SDS-PAGE protein analysis shows that SMc03960 and SMc03960-D27A proteins had been expressed to similar levels (Supplementary Figure S2). Therefore, the residue D27 is required for the release of compounds detected in the spent medium, which is probably due to the thioesterase activity of SMc03960. When a His-tag version of SMc03960 was expressed in *E. coli*, the profile of compounds detected by TLC was similar to that detected for cells expressing SMc03960, indicating that the His-tag does not significantly affect the activity of the protein (Figure 2A).

In order to determine the identity of the spots observed by TLC, lipid extracts from the spent medium of *E. coli* cultures harboring either the *smc03960*-expressing construct pIML50 or the empty vector pET17b (control) were obtained and analyzed by gas chromatography/mass spectrometry (GC/MS) (see Materials and Methods). These analyses revealed the presence of small and similar amounts of 2-TDC and 2-pentadecanone (2-PDC) in the lipid extracts from the control cultures (Figure 2B and 2C). Overexpression of *smc03960* significantly increased the production of 2-TDC and 2-PDC relative to the strain carrying the empty vector pET17b (approximately 55 and 36-fold increase for 2-TDC and 2-PDC, respectively) (Figure 2B). Moreover, 2-undecanone (2-UDC), which was absent in the lipid extracts of control cultures carrying pET17b, could be identified in those of the *smc03960*-overexpressing strain (Figure 2B). These results indicate that SMc03960 participates in MK production. Moreover, the observation that 2-TDC was the predominant MK detected in the overexpressing strain indicates that 3-oxo-myristoyl-ACP and/or 3-oxo-myristoyl-CoA might be the preferred substrates of the thioesterase.

These analyses also revealed an increase in the amount of some free fatty acids in the spent medium of the *smc03960*-overexpressing strain relative to the control strain (Figure 2D), confirming the observations of the TLC analyses (Figure 2A). The largest increase was observed for 3-hydroxy-palmitic acid (3OH-C16:0) followed by 3-hydroxy-myristic acid (3OH-C14:0) (13.5 and 10.5-fold increase, respectively, Supplementary Table S1) suggesting that 3-hydroxy-palmitoyl-ACP/CoA and 3-hydroxy-myristoyl-ACP/CoA might be among the preferred substrates for SMc03960 activity. Furthermore, the expression of *smc03960* in *E. coli* led to a significant increase of the unsaturated fatty acids C12:1 and C14:1 (4.6 and 3.7-fold increase, respectively), and especially of C16:1 and C18:1 (8.6 and 7.3-fold increase, respectively, Supplementary Table S1. Considering the whole pool of unsaturated fatty acids, an overall increase of 4.8-fold was detected in response to *smc03960* expression, whereas the amount of saturated fatty acids only increased by 1.6-fold. Indeed, with the exception of C12:0, no significant increase in the amount of saturated fatty acids was observed (Figure 2D), indicating a preference of the enzyme for unsaturated fatty acids versus saturated fatty acids.

These results indicate that the substrate specificity of the SMc03960 thioesterase is broad in terms of chain length, oxidation state, and saturation level. This characteristic, which has also been observed associated with ALT-like thioesterases from across the plant kingdom [38], could be harnessed for the production of industrially valuable fatty acid and fatty acid-derived products.

### *In vitro* substrate specificity of His-SMc03960

Kinetic constants of purified His-SMc03960 with various acyl-CoA and acyl-AcpP substrates^1^

As described in the previous section, expression of *smc03960* in *E. coli* led to the release of different MKs and fatty acids (Figure 2). The nature of the compounds released as the result of SMc03960 activity depends on the availability of substrates present in the *E. coli* host. To further define the substrate specificity of SMc03960, we decided to evaluate its activity *in vitro*. For that, the protein was overexpressed in *E. coli* with a His-tag at the *N*-terminus and purified to homogeneity (Supplementary Figure S3). For *in vitro* reactions with His-SMc03960, various acyl-CoAs as well as acyl-ACPs derived from purified AcpP of *S. meliloti* were used as substrates. While acyl-CoAs are commercially available, the different acyl-AcpP substrates were synthesized during this study. First, AcpP from *S. meliloti* was obtained in its apo form from an *E. coli* overproducing strain. To obtain holo-AcpP, we used a holo-ACP synthase (AcpS, Supplementary Figure S3) that transfers the 4′-phosphopantetheine group from CoA to apo-AcpP. Acylation of holo-AcpP with specific acyl groups (Table 1, acyl-AcpP substrate) was achieved by using acyl-ACP synthetase from *E. coli* (Aas, Supplementary Figure S3).

**Table:** 

Acyl-CoA substrate	*K*_M_ (µM)	*k*_cat_ (s^-1^) x10^-4^	*k*_cat_/*K*_M_ (s^-1^ M^-1^)
Propionyl-CoA [C_3:0_]	634.9 ± 69	41 ± 1.7	6.5
Octanoyl-CoA [C_8:0_]	96.3 ± 17.8	4.6 ± 0.2	4.8
Decanoyl-CoA [C_10:0_]	147.1 ± 18.3	20 ± 0.7	13.7
Lauroyl-CoA [C_12:0_]	42.6 ± 6	22 ± 0.8	51.8
Myristoyl-CoA [C_14:0_]	18.6 ± 2.2	20.8 ± 0.5	111.9
Palmitoyl-CoA [C_16:0_]	38.5 ± 4.9	23.2 ± 0.8	60.3
**Acyl-AcpP substrate**			
Lauroyl-AcpP [C_12:0_]	37.6 ± 6.2	12.8 ± 0.6	34.0
Myristoyl-AcpP [C_14:0_]	50.5 ± 18.5	69.7 ± 1.3	138.0
Palmitoyl-AcpP [C_16:0_]	135.5 ± 26.8	77.7 ± 8.5	57.3
Stearoyl-AcpP [C_18:0_]	70.8 ± 29.9	76.3 ± 14	107.8
3-hydroxy-lauroyl-AcpP [3OH-C_12:0_]	103.5 ± 13	109.4 ± 4.2	105.3
3-hydroxy-myristoyl-AcpP [3OH-C_14:0_]	66.7 ± 5	250.5 ± 10.1	374.8
3-hydroxy-palmitoyl-AcpP [3OH-C_16:0_]	120.0 ± 9	101.2 ± 9.1	83.4
cis-9-tetradecenoyl-AcpP [cis-9-C_14:1_]	31.3 ± 11	20.3 ± 6.4	65.1
cis-9-hexadecenoyl-AcpP [cis-9-C_16:1_]	71.1 ± 20	30.0 ± 5.2	42.3
cis-9-octadecenoyl-AcpP [cis-9-C_18:1_]	69.0 ± 18.4	87.3 ± 10.9	126.6

1 Data correspond to the average of at least 3 independent measurements ± standard deviation.

**Figure 3:**
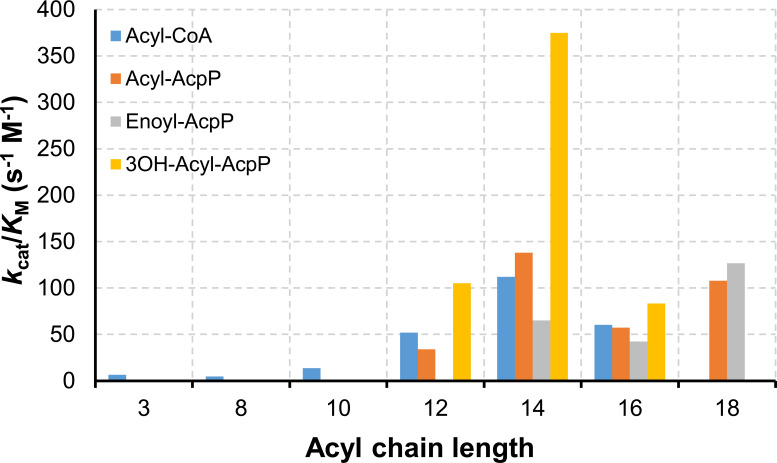
Catalytic efficiency of SMc03960 with different substrates. Specificity constants *k*_cat_/*K*_M_ (s^-1^ M^-1^) of His-SMc03960 for saturated acyl-CoAs (Acyl-CoAs), or for *S. meliloti* AcpPs carrying either saturated fatty acids (Acyl-AcpP), monounsaturated fatty acids (Enoyl-AcpP) or 3-hydroxy fatty acids (3OH-Acyl-AcpP). The X-axis indicates the chain length of the different substrates.

His-SMc03960 showed activity with a broad range of acyl-CoAs and acyl-AcpPs (Table 1). Only minor activity was detected with the short- and medium-chain acyl-CoA substrates propionyl-CoA, octanoyl-CoA, and decanoyl-CoA. Among the tested acyl-CoA substrates, the highest catalytic efficiency was detected for myristoyl-CoA, while efficiency was reduced to about half for lauroyl-CoA and palmitoyl-CoA (Figure 3, Table 1).

Among the saturated acyl-AcpPs tested, the highest thioesterase activity was also found for the C14 chain-length myristoyl-AcpP, and significant activity was detected for the C12:0, C16:0, and C18:0-derived acyl-AcpPs. When a monounsaturated fatty acid of C16 or C18 was attached to AcpP (enoyl-AcpP), the catalytic efficiency of His-SMc03960 was similar to that found for the saturated acyl-AcpPs counterparts. However, the catalytic efficiency of His-SMc03960 with tetradecenoyl-AcpP was half of that found for myristoyl-AcpP. In our *in vitro* assays, His-SMc03960 showed the highest substrate preference for 3-hydroxy-myristoyl-AcpP, while the catalytic efficiency for AcpPs carrying 3-hydroxy fatty acids of C12 or C16 was only a quarter of that observed for 3-hydroxy-myristoyl-AcpP (Figure 3, Table 1). It is noteworthy that the relatively high specificity (*k*_cat_/*K*_M_) for 3-hydroxy-myristoyl-AcpP is mainly due to an elevated turnover number (*k*_cat_) (Table 1).

In summary, the *in vitro* results show that His-SMc03960 can hydrolyze acyl-CoAs and acyl-AcpPs in the range from C12 to C18 and, among the tested substrates, the protein exhibits the highest activity with 3-hydroxy-myristoyl-AcpP followed by myristoyl-AcpP with 38% of the activity found for the hydroxylated form. Recently, the presence of the 31-CQH[G/C]RH-36 motif on the central α-helix of plant ALT-type thioesterases was associated with substrate specificity and specifically with preference for C12–C14 3-hydroxy-acyl- and 3-oxo-acyl-ACP substrates [38]. Despite exhibiting a similar broad substrate specificity, SMc03960 lacks such a motif (Figure 1A). Whether this difference allows the rhizobial thioesterase to accommodate longer acyl chains and/or acyl-CoA substrates awaits investigation.

The high specificity for 3-hydroxy-acyl-AcpPs correlates with the increased accumulation of 3OH-C14:0 and 3OH-C16:0 fatty acids observed in the spent media of *E. coli* strains overexpressing SMc03960. In the spent medium, a higher accumulation of unsaturated fatty acids compared with that of saturated fatty acids was observed (Figure 2D, Supplementary Table S1). However, His-SMc03960 did not show a preference for the enoyl-AcpPs tested with respect to the saturated acyl-AcpP counterparts (Table 1). The discrepancy concerning unsaturated fatty acids between the *in vivo* and *in vitro* data could be due to differences in the diffusion rate from the cell to the spent medium between saturated and unsaturated fatty acids. Other possibilities are: i) His-SMc03960 might show different specificities for unsaturated fatty acids than native SMc03960; ii) unsaturated fatty acids accumulated in the spent medium might be derived from acyl-CoAs; iii) the unsaturated species detected in the spent medium might be different from the unsaturated species tested in our *in vitro* assays (i.e. unsaturation present in another position); or iv) free saturated fatty acids might be better degraded by β-oxidation than the unsaturated ones.

Although acyl-CoA thioesterase activity has been described for different YbgC-like proteins [27–29], and acyl-ACP thioesterase activity has been proposed for *E. coli* YbgC based on its binding to ACP [32], this is the first time that acyl-ACP thioesterase activity has been demonstrated for a YbgC-like protein.

### *In vitro* formation of 2-TDC by His-SMc03960

**Figure 4:**
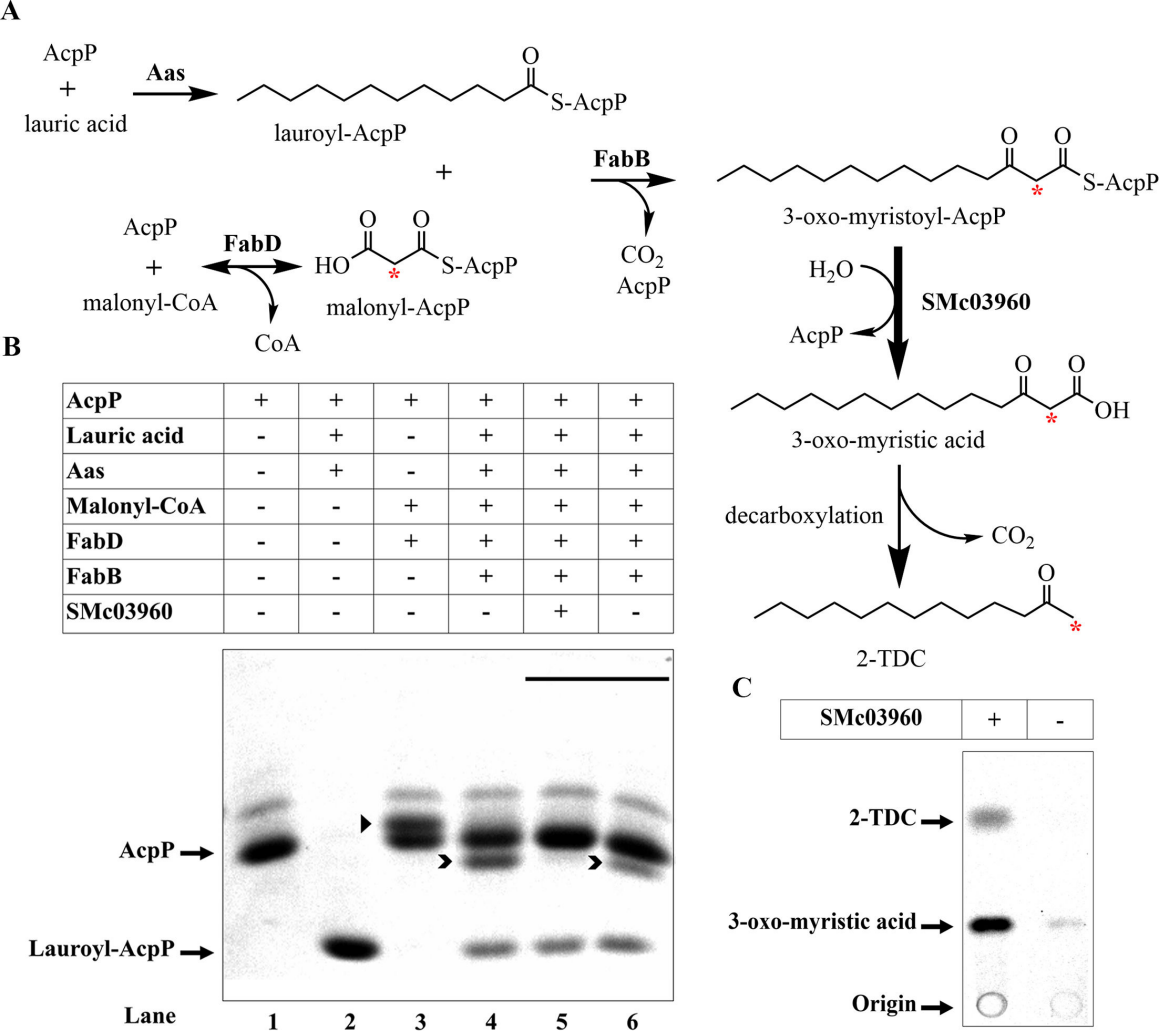
*In vitro* biosynthesis of 2-tridecanone by SMc03960 using the substrate 3-oxo-myristoyl-AcpP. (**A**) Reactions and enzymes leading to 2-tridecanone (2-TDC) synthesis: formation of lauroyl-AcpP by Aas and of malonyl-AcpP by FabD; condensation of lauroyl-AcpP and malonyl-AcpP by FabB leads to 3-oxo-myristoyl-AcpP; SMc03960 can hydrolyze 3-oxo-myristoyl-AcpP to 3-oxo-myristic acid which via spontaneous decarboxylation is converted into 2-TDC. The carbons labeled when using [2-^14^C]malonyl-CoA are marked with red asterisks. (**B**) Analysis of the consecutive AcpP intermediates by conformationally sensitive PAGE. The positions of AcpP and lauroyl-AcpP on the gel are indicated. A triangle points to malonyl-AcpP and arrow heads indicate the position of 3-oxo-myristoyl-AcpP. The solid bar over the gel highlights samples that had been incubated with His-SMc03960 or with buffer only. (**C**) Radio-TLC analysis of lipidic products obtained after using [2-^14^C]malonyl-CoA in the reactions to obtain 3-oxo-myristoyl-AcpP and then treated with purified SMc03960 (+) or only with buffer (-). The positions of 2-TDC and possible 3-oxo-myristic acid are indicated.

The MK 2-TDC was found to be the most abundant MK after expression of *smc03960* in *E. coli* (Figure 2, Supplementary Table S1). The tomato thioesterase MKS2, which shows homology to SMc03960, uses the intermediate of fatty acid biosynthesis 3-oxo-myristoyl-ACP to form 3-oxo-myristic acid that, after decarboxylation, leads to 2-TDC formation [22]. We decided to test whether, like MKS2, purified His-SMc03960 was capable of generating 2-TDC *in vitro* by using 3-oxo-myristoyl-AcpP as a substrate. To obtain 3-oxo-myristoyl-AcpP, a series of reactions were performed as outlined in Figure 4A. The substrates lauroyl-AcpP and malonyl-AcpP were obtained by using the enzymes Aas from *E. coli* and His-FabD from *S. meliloti* (FabD) (Supplementary Figure S3), respectively. To follow the formation of the respective acyl-AcpPs, conformationally sensitive PAGE was used and proteins were visualized by Coomassie blue staining (Figure 4B). Modification of holo-AcpP (Figure 4B, lane 1) with lauric acid through the action of Aas results in a faster migrating band that corresponds to lauroyl-AcpP (Figure 4B, lane 2). The reaction catalyzed by FabD is reversible, and modification of AcpP with malonyl-CoA in the presence of FabD results in a 50% conversion, with malonyl-AcpP corresponding to the upper migrating band (Figure 4B, lane 3). After incubation of lauroyl-AcpP and malonyl-AcpP with His-FabB of *S. meliloti* (FabB), the band corresponding to malonyl-AcpP disappeared, whereas a new band was detected that possibly corresponds to 3-oxo-myristoyl-AcpP (Figure 4B, lane 4). Because of the unstable nature of 3-oxo-myristoyl-AcpP, it was not further purified, and the reaction mixture was used for *in vitro* thioesterase activity assays. Incubation for 30 min of this sample with His-SMc03960 led specifically to the loss of the band identified as 3-oxo-myristoyl-AcpP, while treatment under the same conditions with buffer only did not result in the disappearance of the band (Figure 4B, lanes 5 and 6). The amount of lauroyl-AcpP detected in these two reactions was not significantly affected, indicating that His-SMc03960 has a preference to hydrolyze 3-oxo-myristoyl-AcpP over lauroyl-AcpP. To detect the possible formation of 2-TDC by TLC, radiolabeled [2-^14^C]malonyl-CoA was used in the reaction with FabD to form [2-^14^C]malonyl-AcpP. The use of this radiolabeled precursor and lauroyl-AcpP leads to [2-^14^C]3-oxo-myristoyl-AcpP in the reaction catalyzed by FabB and [2-^14^C]3-oxo-myristic acid after hydrolysis by His-SMc03960. Spontaneous decarboxylation of the unstable compound [2-^14^C]3-oxo-myristic acid leads to [1-^14^C]2-TDC (Figure 4A). After direct extraction of the lipidic compounds of the reaction containing [2-^14^C]3-oxo-myristoyl-AcpP and treated with His-SMc03960, a major spot was observed, which probably corresponds to 3-oxo-myristic acid, while only a faint band was observed in the reaction incubated with buffer only. Furthermore, a radiolabeled band that migrates as the 2-TDC standard was only observed in the sample treated with His-SMc03960 (Figure 4C). Lack of any treatment with heat and/or acid [22] may have prevented a further conversion of 3-oxo-myristic acid to 2-TDC. These data demonstrate that His-SMc03960 is able to hydrolyze 3-oxo-myristoyl-AcpP to 3-oxo-myristic acid, which can spontaneously decarboxylate to 2-TDC, thereby confirming the role of the YbgC-like protein SMc03960 in 2-TDC formation. Interestingly, the spot identified as 3-oxo-myristic acid in Figure 4C migrates like the unidentified product (U) observed after *in vivo* expression of SMc03960 (Figure 2A). Therefore, we tentatively assign the unknown spot in Figure 2A as a mixture of 3-oxo-acids, namely 3-oxo-lauric acid, 3-oxo-myristic acid, and 3-oxo-palmitic acid which, after decarboxylation, would lead to the observed MKs, 2-UDC, 2-TDC, and 2-PDC, respectively (Figure 2, Supplementary Table S1). Overall, our *in vitro* results show that SMc03960 has preference for 3-hydroxy- and 3-oxo-myristoyl-ACP substrates.

### Overexpression of *smc03960* increases the production of volatile 2-TDC in wild type and *fadD* mutant strains of *S. meliloti*

**Figure 5:**
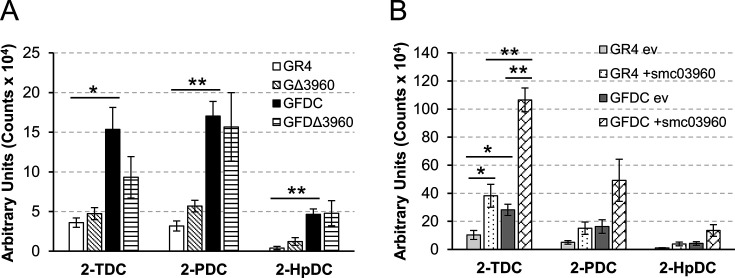
Effect of *smc03960* loss-of-function (A) and overexpression (B) on methylketone production in *S. meliloti.* The levels of methylketones emitted by different *S. meliloti* strains into the headspace were determined by Solid Phase MicroExtraction-GC/MS. Production of the methylketones 2-tridecanone (2-TDC), 2-pentadecanone (2-PDC), and 2-heptadecanone (2-HpDC) in (A) *S. meliloti* wild type strain GR4 (GR4), and derivatives mutated in *smc03960* (GΔ3960), in *fadD* (GFDC) or mutated in both *smc03960* and *fadD* (GFDΔ3960), and in (B) *S. meliloti* wild type strain GR4 (GR4) and its *fadD* derivative mutant (GFDC) carrying either an empty vector (ev), or an *smc03960*-expressing plasmid (+smc03960). Values represent the mean peak area and standard error obtained in at least three independent experiments. Significant differences according to a pairwise ANOVA test are indicated (*P<0.05, **P<0.005).

To test whether SMc03960 plays a role in the generation of MKs in *S. meliloti*, we obtained *smc03960* mutants and *smc03960*-overexpressing strains derived from both the wild type and *fadD* mutant genetic backgrounds. By constructing *fadD*-derivative strains with altered *smc03960* expression, we pursue to confirm *in vivo* the thioesterase activity of SMc03960 on acyl-ACP intermediates. Volatiles emitted by the wild type and *fadD* mutant strains in which the *smc03960* gene was either inactivated or overexpressed were analyzed by solid phase microextraction (SPME)-GC/MS after growth on MM (1% agar) for 48 hours. As shown in Figure 5A and as previously reported [11], the wild type strain GR4 produces the MKs 2-TDC, 2-PDC, and 2-heptadecanone (2-HpDC), and *fadD* loss-of-function in strain GFDC leads to increased production of these volatiles (increases of approximately 4, 5, and 11-fold for 2-TDC, 2-PDC, and 2-HpDC, respectively) (Figure 5A).

Deletion of *smc03960* in a wild type background (strain GΔ3960) did not cause any significant effect on MK production compared with the wild type strain GR4 (Figure 5A). Likewise, and despite a small but statistically not significant reduction in 2-TDC levels, deletion of *smc03960* in a *fadD* mutant background (strain GFDΔ3960) did not affect the emission of volatile MKs (Figure 5A). In contrast, overexpression of *smc03960* conferred by the presence of the pIML55 plasmid increased approximately 3.7-fold the levels of 2-TDC emitted by both the wild type and the *fadD* mutant strains compared with those emitted by the same strains carrying the empty vector pNG28 (Figure 5B). Overexpression of *smc03960* also seemed to induce small increases in the levels of the other two MKs (2-PDC and 2-HpDC) produced by the wild type and the *fadD* mutant, but in these latter cases, the differences were not statistically significant. These results indicate that the thioesterase activity of SMc03960 can contribute to 2-TDC production in *S. meliloti*. Moreover, the increased emission of 2-TDC observed in the *smc03960*-overexpressing *fadD* mutant suggests that SMc03960 can use 3-oxo-myristoyl-AcpP as a substrate, as shown *in vitro* using purified His-SMc03960 (Figure 4). However, the fact that deletion of *smc03960* does not abolish MK production is indicative that additional yet unknown enzymatic activities participate in MK synthesis in *S. meliloti*. In addition to SMc03960, *S. meliloti* contains several ORFs with a thioesterase domain (Supplementary Table S2). However, at this point, it is not known which of them, besides SMc03960, might be involved in MK production.

MKS1 from tomato is a decarboxylase that facilitates the conversion of 3-oxo-acids released by MKS2 to MKs [22]. Although SMc03960 is homologous to MKS2, no protein with significant similarity to MKS1 was found in *S. meliloti* proteomes. At present, it is not known whether an enzyme exists in *S. meliloti*, without homology to MKS1, that catalyzes decarboxylation of 3-oxo-acids, or if formation of MKs depends on spontaneous decarboxylation only.

Expression of *smc03960* in *S. meliloti* strains only led to a significant increase of 2-TDC formation (Figure 5B). However, while 2-TDC was the most abundant MK after expression of *smc03960* in *E. coli*, significant amounts of 2-UDC and 2-PDC were also formed (Figure 2B). These findings could be due to the different techniques and growth conditions used for detection and/or to a possible impact of the AcpP sequences of each bacterium over the specificity of SMc03960, as recently described for the acyl-ACP thioesterase *Ch*FatB2 [39].

## Conclusions

This study uncovers and provides functional insights about a bacterial fatty acyl-ACP/CoA thioesterase enzyme with broad substrate specificity (C12 to C18), which leads to the formation of fatty acids, 3-hydroxy fatty acids, and several MKs, including 2-TDC. Enzymes for the biosynthesis of MKs have been identified in different plants, including MKS2, which is the MK synthase from wild tomato. However, information about bacterial thioesterases involved in MK production is rather limited. Here, we identify SMc03960 from the bacterial legume endosymbiont *S. meliloti* as a Tol-Pal associated YbgC-like thioesterase and an MKS2 homolog that leads mainly to the formation of MKs and 3-hydroxy fatty acids when heterologously expressed in *E. coli*. In enterobacteria, data suggest the joint participation of YbgC and the Tol-Pal system in phospholipid metabolism/homeostasis [32,40]. Additional research efforts are needed to elucidate whether SMc03960 plays a similar role together with the Tol-Pal apparatus in *S. meliloti*. Using a combination of *in vivo* and *in vitro* approaches, we showed that SMc03960 is able to hydrolyze acyl-CoAs and acyl-ACPs of different chain length, oxidation state, and saturation level. Furthermore, the pathway for 2-TDC production was reconstructed *in vitro* by treatment of synthesized 3-oxo-myristoyl-ACP with purified His-SMc03960. This is the first time that both MK production and acyl-ACP thioesterase activity have been demonstrated for a bacterial YbgC-like protein. SMc03960 is not essential for MK production in *S. meliloti*. However, in line with its role in 2-TDC production, we show that ectopic expression of *smc03960* in the alfalfa endosymbiont increases significantly the formation of 2-TDC. The physiological role of SMc03960 in *S. meliloti,* including a putative functional association with the Tol-Pal system, as well as the identification of other enzymatic activities involved in MK production in this bacterium, awaits further investigations. Meanwhile, the broad substrate specificity of SMc03960 could be harnessed for the production of industrially valuable fatty acid and fatty acid-derived products.

## Materials and methods

### Bacterial strains, plasmids, and growth conditions

Bacterial strains and plasmids used in this work and their relevant characteristics are listed in Supplementary Table S3. *E. coli* strains were grown in Luria–Bertani (LB) medium [41] or in M9 minimal medium [42] at 37°C or at 30°C when sinorhizobial proteins were expressed. *S. meliloti* strains were grown at 28°C either in complex tryptone yeast (TY) medium [43], or in Robertsen minimal medium (MM) [44]. When required, antibiotics were added at final concentrations of: 200 μgˑml^-1^ ampicillin, 100 μgˑml^-1^ carbenicillin, 20 μgˑml^-1^ chloramphenicol, 50 μgˑml^-1^ kanamycin, 50 μgˑml^-1^ streptomycin, 100 μgˑml^-1^ spectinomycin, 10 μgˑml^-1^ tetracycline for *E. coli* and 200 μgˑml^-1^ kanamycin, 200 μgˑml^-1^ streptomycin, 100 μgˑml^-1^ spectinomycin, and 10 μgˑml^-1^ tetracycline for *S. meliloti*. To improve reproducibility, all liquid cultures of *S. meliloti* were initiated from glycerol stocks. Plasmids were mobilized into *S. meliloti* by diparental mating using the *E. coli* S17-1 donor strain as previously described [45].

### DNA manipulations

Recombinant DNA techniques were performed according to standard protocols [41]. Commercial sequencing of amplified genes by Eurofins Medigenomix (Martinsried, Germany) corroborated correct DNA sequences. Oligonucleotide primer sequences are listed in Supplementary Table S4.

### Construction of expression plasmids

Using PCR and specific oligonucleotides 186 and 187 (Supplementary Table S4), *smc03960* was amplified from *S. meliloti* genomic DNA and cloned into pET9a previously digested with *Nde*I and *Bam*HI resulting in plasmid pDJ1. Subsequently, *smc03960* was obtained from pDJ1 as a *Nde*I-*Bam*HI fragment and subcloned either into pET17b digested with *Nde*I-*Bam*HI (resulting in plasmid pIML50) or into pET16b digested with *Nde*I-*Bam*HI (resulting in plasmid pGRH01). For use in *S. meliloti*, pIML50 was linearized with *Bam*HI and ligated into *Bam*HI-linearized pRK404 resulting in pIML55. To obtain a plasmid for expression of His-FabB, the gene *smc00327* (*fabB*) was PCR amplified from *S. meliloti* genomic DNA using specific oligonucleotides (Supplementary Table S4) and cloned into pET16b digested with *Nde*I and *Bam*HI, resulting in plasmid pCSB10.

In order to exchange the putative aspartic acid active site (D27) of SMc03960 by an alanine, plasmid pIML50 was used as template for site-directed mutagenesis. For the construction of the mutation, the QuikChange II XL Site-Directed Mutagenesis Kit (Agilent Technologies) and the primers a80c_FW and a80c_R (Supplementary Table S4), which were designed using the QuikChange Primer Design Program available online (www.genomics.agilent.com/primerDesignProgram.jsp), were used. The mutation in the resulting plasmid pGRHD27A was confirmed by sequencing.

### Construction of *S. meliloti* mutants

The GFDC mutant was obtained by allelic exchange using construct pK18fadDCKm as previously described for the Rm1021 *fadD* mutant [7]. To obtain *S. meliloti smc03960* mutants, a version of the gene was created by deleting most of the coding sequence (409 bp deletion) and inserting a streptomycin/spectinomycin (Sm/Sp) resistance cassette. For that, DNA fragments of approximately 1100 bp corresponding to the upstream and downstream regions of *smc03960* were PCR amplified using *S*. *meliloti* genomic DNA and primer pairs 75/76 and 77/78, respectively (Supplementary Table S4). The resulting PCR products were subcloned into pBluescript KS(+) to obtain pBSΔ3960. Next, the Sm/Sp resistance cassette from pHP45Ω was inserted into a unique *Bam*HI site created within *smc03960* to obtain pBSΔ3960SS. The *Eco*RI-*Xba*I fragment containing the disrupted version of *smc03960* was cloned into the suicide vector pK18*mobsacB* to obtain pK18Δ3960SS. This plasmid was introduced into GR4 and GFDC via conjugation with the *E. coli* strain S17-1, and allele replacement events were selected by Sm/Sp resistance and sensitivity to sucrose. The resulting mutants were confirmed by Southern hybridization with a specific probe.

### *In vivo* labeling of *E. coli* with ^14^C-acetate and analysis of lipid extracts by TLC

The lipid composition of different *E. coli* strains was determined following labeling with [1-^14^C]acetate (specific activity: 55 mCi/mmol; PerkinElmer) as described previously [10]. Lipids from spent media were extracted with 0.4 volumes of acidified ethyl acetate (0.01% acetic acid). The lipids obtained were analyzed by TLC using high-performance TLC silica gel 60 plates (Merck) and hexane: ethyl acetate: acetic acid (70:30:4 [vol/vol/vol]) as mobile phase. Radioactivity was detected using a Storm 820 PhosphorImager (Amersham Biosciences). Image analysis was carried out using ImageQuant TL (Amersham Biosciences). *E. coli* BL21(DE3) x pLysS derived strains were grown in M9 minimal medium and protein expression was induced by the addition of 0.1 mM IPTG during the mid-exponential phase of bacterial growth (OD_620nm_ = 0.4). Cultures were collected 4 h after induction with IPTG. For each strain, labeling experiments were repeated at least 3 times, and representative TLCs are shown.

### Analyses of lipid extracts by GC/MS

Cultures of *E. coli* strain BL21(DE3) x pLysS carrying either pIML50 or pET17b (control) were grown at 30°C in 100 ml of M9 minimal medium containing the required antibiotics. Protein expression was induced as described above and cell-free culture supernatants were collected 4 h after induction with IPTG. Prior to extraction, tridecanoic acid methyl ester (10 ppm) (Cromlab S.L., Spain) was added as an internal standard and cell-free spent media were extracted twice with 0.4 volumes of acidified ethyl acetate. The extracts were dried by rotary evaporation at 45°C and dissolved in 1 ml of acidified ethyl acetate. Samples were analyzed after derivatization with N,O-bis (trimethylsilyl) trifluoroacetamide and trimethylchlorosilane (99:1 v/v). Fifty µl of sample were mixed with 50 µl of silylation reagent for 30 minutes at 60°C. After cooling, samples were kept at room temperature for 1 hour prior to injection. GC/MS analyses were performed with an ion trap mass spectrometer, model Varian 450GC 240 MS with a TG5SILMS fused silica capillary column (30 m length, 0.25 mm inner diameter, 0.25 µm film thickness; Thermo Scientific); 1 µl injections were performed by a model CombiPal (CTC Analytic) autosampler. The GC oven was programmed from 40°C (held for 3 min) to 140°C at 15 °C min^-1^. Temperature was held for 2.5 min and then increased up to 300°C at 15 °C min^-1^ and held for 3 min. The injection port temperature was 280°C, and the transfer line temperature was 280°C. The carrier gas, ultra-high purity helium, flowed at a constant rate of 1 ml min^-1^. Injections were splitless, with the split turned on after 0.1 min. For full-scan data acquisition in electron ionization mode (EI), the MS scanned from 40 to 600 atomic mass units at a rate of 0.75 scans per second. Estimation of the relative amounts of MKs, fatty acids, and fatty acid methyl esters (FAMEs) was calculated by comparing the areas under the peaks on the chromatograms in the selected ion monitoring (SIM) mode to the area under the peak of the internal standard (FAME C13:0). For 2-undecanone (2-UDC), 2-TDC, and 2-pentadecanone (2-PDC), external standard quantification (*m/z* 58 areas) was performed with authentic standards. Fatty acid species were identified using retention times and mass spectral information.

### Analyses of volatile MKs by SPME-GC/MS

These analyses were performed as previously described [11]. Briefly, ten µl of washed, 10-fold-concentrated *S. meliloti* cultures grown in TY broth to the late exponential phase were inoculated onto the surface of 3 ml of MM (1% agar) in 10 ml glass vials and incubated at 30°C for 2 days. To collect volatiles from the headspace of the vials, an automatic CombiPal-SPME system attached to the GC/MS (Varian 450GC 240 MS, Ion Trap) was used. The SPME fiber (57298 U, Supelco) was conditioned for 1 h at 250°C in a stream of helium and then introduced into the headspace of the vials at 30°C during 60 minutes and desorbed at 280°C (6 min). Samples were separated in a DB5MS-UI column (30 m, 0.25 mm inside diameter, 0.25 μm, 122–5532UIE, Agilent Technologies). The protocol program used for oven temperature was programmed from 40°C (held for 3 min) to 140°C at 15 °C min^-1^. Temperature was held for 2.5 min and then increased up to 300°C at 15 °C min^-1^ and held for a further 3 min. Helium was used as the carrier gas, with a flow rate of 1 ml min^-1^. The mass spectrometer was run at 220°C operated in the positive electron ionization mode at 70 eV. The mass spectra of detected peaks were compared with those in the NIST database. The production of the different MKs was further confirmed by comparison with pure standards which were prepared and analyzed in the same manner as the biological samples.

### Overexpression of proteins in *E. coli* and cell-free crude extract preparation

*E. coli* BL21(DE3) x pLysS harboring additionally the desired plasmid for protein overexpression was grown at 30°C in LB medium supplemented with chloramphenicol plus the appropriate other antibiotic. At OD_620_ = 0.4, IPTG was added to a final concentration of 0.1 mM. After 4 h (or 2 h for His-FabD and His-FabB) of induction, cells were harvested by low-speed centrifugation at 4°C and cell pellets were resuspended in ice-cold buffer. Cell suspensions were broken by three passages through a French pressure cell at 20,000 pounds per square inch (psi). Broken cell suspensions were centrifuged for 10 min at 5,000* **g*** and the supernatants were recovered except for AcpS, which was overproduced as inclusion bodies, and the pellet was collected.

### Protein purification

Apo-AcpP of *S. meliloti* was obtained from a 1 l culture of the strain BL21(DE3) x pLysS pTB5035 and the cell pellet was resuspended in 20 ml of 50 mM Tris/HCl, pH 6.8 (buffer A). Cold 2-propanol was added dropwise to the cell-free protein extracts until a final concentration of 50% (v/v) was reached. After incubation for 60 min on ice, the precipitate was removed by centrifugation at 15,550 ***g*** for 15 min at 4°C. The supernatant was dialyzed twice against 2 l of buffer A and then applied to a 30 ml DEAE-52 cellulose (Whatman) column. The column was washed with 60 ml buffer B (10 mM BisTris/HCl pH 6, 1 mM CHAPS). Elution was performed with a linear gradient from 0 to 1 M NaCl in buffer B in a total volume of 200 ml. Fractions of 3 ml were collected and analyzed by 20% native PAGE. Fractions containing apo-AcpP were concentrated by ultrafiltration with Amicon® Ultra-4 centrifugal filters (Millipore) at 4°C, the buffer exchanged to 10 mM Tris/HCl, pH 8.0, and kept frozen at −20°C.

In the case of the AcpS protein, the pellets containing inclusion bodies of AcpS were washed twice in 50 mM Tris/HCl, pH 6.8 and after dissolving them in 9.5 M urea, the concentration of urea was slowly diluted to 0.9 M by adding 50 mM Tris/HCl, pH 6.8 buffer. The resulting sample was centrifuged and stored at −20°C in 0.9 M urea, 50 mM Tris/HCl, pH 6.8.

To obtain a fraction of enriched acyl-ACP synthetase (Aas), cell-free crude extract of BL21(DE3) x pLysS containing plasmid pAasH was first subjected to ultracentrifugation at 184.000 *g* for 1 h. The resulting pellet was homogenized in 50 mM Tris/HCl, pH 8, 20 mM MgCl_2_, 2% Triton^TM^ X-100 (Sigma), and after a subsequent ultracentrifugation, the supernatant was aliquoted and stored at −80°C for use in acylation reactions.

To obtain His-SMc03960, the cell-free extract derived from 750 ml culture of the strain BL21(DE3) x pLysS pGRH01 was brought to 100 ml in buffer 50 mM Tris/HCl pH 8.8, 0.5 M urea and was applied to a 5 ml Ni^2+^ agarose column (HiTrap, Pharmacia Biotech, Uppsala, Sweden). After a washing step with 50 mM Tris/HCl pH 8.8, 0.5 M urea, 75 mM imidazole, His-SMc03960 was eluted from the column with 25 ml of 50 mM Tris/HCl pH 8.8, 0.5 M urea, 75 mM EDTA. After elution, proteins were concentrated and buffer was exchanged by means of Amicon® Ultra-4 centrifugal filters (Millipore) to storage buffer consisting of 50 mM Tris/HCl pH 8.8, 0.25 M urea, 10% glycerol. Purified His-SMc03960 was aliquoted and kept frozen at −20°C.

Cells derived from 250 ml culture of strains producing His-FabD or His-FabB, BL21(DE3) x pLyS carrying additionally pSBD02 or pCSB10, respectively, were resuspended in 20 mM sodium phosphate, 0.5 M NaCl, 10 mM imidazole, pH 7.4 and the cell-free crude extracts obtained from them were applied to a 1 ml Ni^2+^ agarose column (HiTrap, Pharmacia Biotech, Uppsala, Sweden). Ni^2+^ chromatography was performed according to the manufacturer’s instructions. Immediately after elution with PBS buffer containing 500 mM imidazole, the buffer was changed to storage buffer (20 mM Na-phosphate buffer pH 7.4, 500 mM NaCl, 10% glycerol) using Amicon® Ultra-4 10 K centrifugal filters. Samples were aliquoted and kept frozen at −20°C.

### Analysis of proteins

AcpP-proteins were analyzed by 20% native PAGE [46] or by conformation-sensitive gels [47] containing 16.8% polyacrylamide in the separation gel and 3.2 M urea in both the separation and stacking gel. Samples were mixed with 6 x application buffer (375 mM Tris/HCl pH 6.8, 60% glycerol, 0.15% bromophenol blue) before they were loaded onto polyacrylamide gels. Gels were stained with Coomassie blue. Protein concentrations were determined with Pierce^TM^ 660 nm Protein assay (Thermo Scientific) using BSA (Sigma) as standard.

### Preparation of acyl-AcpP substrates

First, apo-AcpP was converted to holo-AcpP similarly as previously described [48] by the incubation of apo-AcpP with CoA in the presence of *S. meliloti* AcpS. Reactions were performed in 10 mM Tris/HCl, pH 8.0, containing 0.3 mM CoA, 5 mM MgCl_2_, 10 mM DTT, 30 to 120 μM apo-AcpP and 2.5 μM AcpS. Reactions were incubated at 30°C with shaking (250 rpm) for 5 h. Different acyl-AcpP substrates were prepared using solubilized membranes containing acyl-ACP synthetase (Aas) from *E. coli* [49]. Acylation reactions were carried out as previously described [50] using per reaction as enzymatic source 50 µl of solubilized membranes (1.8 mg protein/ml) of the *E. coli* strain overproducing Aas. The reaction proceeded for 2.5 h at 37°C with shaking (250 rpm). The obtained acyl-AcpPs were purified over 1 ml DEAE-52 cellulose (Whatman) columns by anion exchange chromatography.

### Determination of reaction kinetics of His-SMc03690 for the substrates acyl-CoA and acyl-AcpP

Acyl-CoA substrates were purchased from Sigma (U.S.A.). Thioesterase activity of the different acyl-CoAs was measured spectrophotometrically in triplicates in a 96-well plate reader at 30°C by monitoring the formation of a complex of the released CoA with 5,5′-dithio-bis(2-nitrobenzoic acid) (DTNB, Ellman’s reagent). The absorbance of the resulting complex was measured at 412  nm. Reactions were initiated by the addition of purified His-SMc03960 (5 μM final concentration) to wells containing 50  mM Tris/HCl pH 7.5, 100  μM DTNB, and the acyl-CoA substrates (ranging from 0 to 300  μM). Initial velocity rates were plotted versus substrate concentration, analyzed for non-linear regression calculations using the Michaelis–Menten equation *V_0_ = V*_max_[*S*]/ ([*S*]  +  *K*_M_), by means of the Origin 8.0 (Microcal) software, allowing determination of maximal velocity (*V*_max_) and Michaelis–Menten constant (*K*_M_). Turnover (*k*_cat_) could then be obtained using the equation *k*_cat_ *= V*_max_/[*E*].

The activity of His-SMc03960 over different acyl-AcpPs was determined by analysis on conformationally sensitive gels (16.8% polyacrylamide, 3.2 M urea) of the amount of holo-AcpP released after treatment of the respective acyl-AcpPs for 0, 10, 20, 30, or 40 mins. Reactions were performed at 30°C in 50  mM Tris/HCl pH 7.5 using 5 μM purified His-SMc03960 and acyl-AcpPs in the concentration ranges specified in Table 2. ImageLab software version 5.2.1 (Bio-Rad) was used for image acquisition and densitometric analysis of the gels. Kinetics constants were calculated as for the acyl-CoA substrates.

**Table 2:** Concentration ranges for different acyl-AcpPs used for kinetic determinations.

Acyl-AcpPs substrate	Concentration range (µM)
C12:0-AcpP	0–240
C14:0-AcpP	0–200
C16:0-AcpP	0–250
C18:0-AcpP	0–140
C12-3OH-AcpP	0–310
C14-3OH-AcpP	0–310
C16-3OH-AcpP	0–310
C14:1-AcpP	0–100
C16:1-AcpP	0–125
C18:1-AcpP	0–170

### Substrate preparation and enzyme assays for 2-TDC formation with His-SMc03960

To obtain the substrate [^14^C]3-oxo-myristoyl-AcpP for SMc03960 thioesterase activity assays, three enzymatic reactions are required (Figure 4). In the first reaction, 12:0 lauric acid (Sigma) is converted to lauroyl-AcpP using holo-AcpP from *S. meliloti* and Aas of *E. coli* as described under ‘Preparation of acyl-AcpP substrates’. Next, we obtained [2-^14^C]malonyl-AcpP using [2-^14^C]malonyl-CoA (specific activity 26.5 mCi mmol^−1^; American Radiolabeled Chemicals) in the presence of His-FabD as previously described [48]. After 30 min of incubation of the vial containing the malonyzation reaction, His-FabB and lauroyl-AcpP at 5 µM and 10 µM final concentrations were added, and the reaction was incubated for 1 h at 30°C. The reaction was divided into two equal parts, and to one His-SMc03960 to 5 µM final concentration was added and to the other buffer only. After 30 min incubation at 30°C, reactions were extracted with *n*-hexane and after concentration, the organic phase was analyzed by TLC. To visualize conformation changes of AcpP provoked by different acyl groups, similar reactions were carried out except that no radioactivity was used. Samples at the different steps were mixed with 6 x application buffer (375 mM Tris/HCl pH 6.8, 60% glycerol, 0.15% bromophenol blue) and analyzed by conformationally sensitive gels (16.8% polyacrylamide, 3.2 M urea) [47].

## Supplementary material

Online supplementary material 1

## Data Availability

Supporting data are included within the main article and its supplementary file. The authors of this manuscript agree to make any materials, data, code, and associated protocols available upon request.

## Abbreviations

ACP: acyl carrier protein
ALT: acyl-lipid thioesterase
CoA: coenzyme A
EI: electron ionization mode
FAME: fatty acid methyl ester
GC/MS: gas chromatography/mass spectrometry
2-HpDC: 2-heptadecanone
IPTG: isopropyl β-D-1-thiogalactopyranoside
MK: methylketone
MKS2: methylketone synthase 2
MM: minimal medium
OD: optical density
3OH-FA: 3-hydroxy fatty acid
PAGE: polyacrylamide gel electrophoresis
PCR: polymerase chain reaction
2-PDC: 2-pentadecanone
SDS: sodium dodecyl sulfate
SPME: solid phase microextraction
2-TDC: 2-tridecanone
TE: thioesterase
TY: tryptone/yeast extract medium
2-UDC: 2-undecanone
3oxo-FA: 3-oxo fatty acid
