# Supplementary material for: Identification of a Sinorhizobium meliloti YbgC-like thioesterase that contributes to the production of the infochemical 2-tridecanone
Source: Biochem J. 2025 Sep 22;482(19):1375–91. doi: 10.1042/BCJ20253120 (PMC12794305; doi:10.1042/BCJ20253120)
Supplement: Online supplementary material 1 [file bcj-482-19-BCJ20253120-s001.docx]

**Supplementary Material**

**Identification of a *Sinorhizobium meliloti* YbgC-like thioesterase that contributes to the production of the infochemical 2-tridecanone**

**Lydia M. Bernabéu-Roda^a#^, Geovanny Rivera-Hernández^b#^, Virginia Cuéllar^a^, Rafael Núñez^c^,** **Ángeles Moreno-Ocampo^b^, Christian Sohlenkamp^b^, Otto Geiger^b^, María J. Soto^a*^, Isabel M. López-Lara^b*^**

^a^ Department of Biotechnology and Environmental Protection, Estación Experimental del Zaidín, CSIC, 18008 Granada, Spain

^b^ Programa de Ecología Genómica, Centro de Ciencias Genómicas, Universidad Nacional Autónoma de México, Cuernavaca, Morelos, C.P. 62210, Mexico

^c^ Scientific Instrumentation Service, Estación Experimental del Zaidín, CSIC, 18008 Granada, Spain

****Correspondence to María J. Soto and Isabel M. López-Lara:*** *mariajose.soto@eez.csic.es (M.J. Soto), isabel@ccg.unam.mx (I.M. López-Lara)*

^#^These authors contributed equally


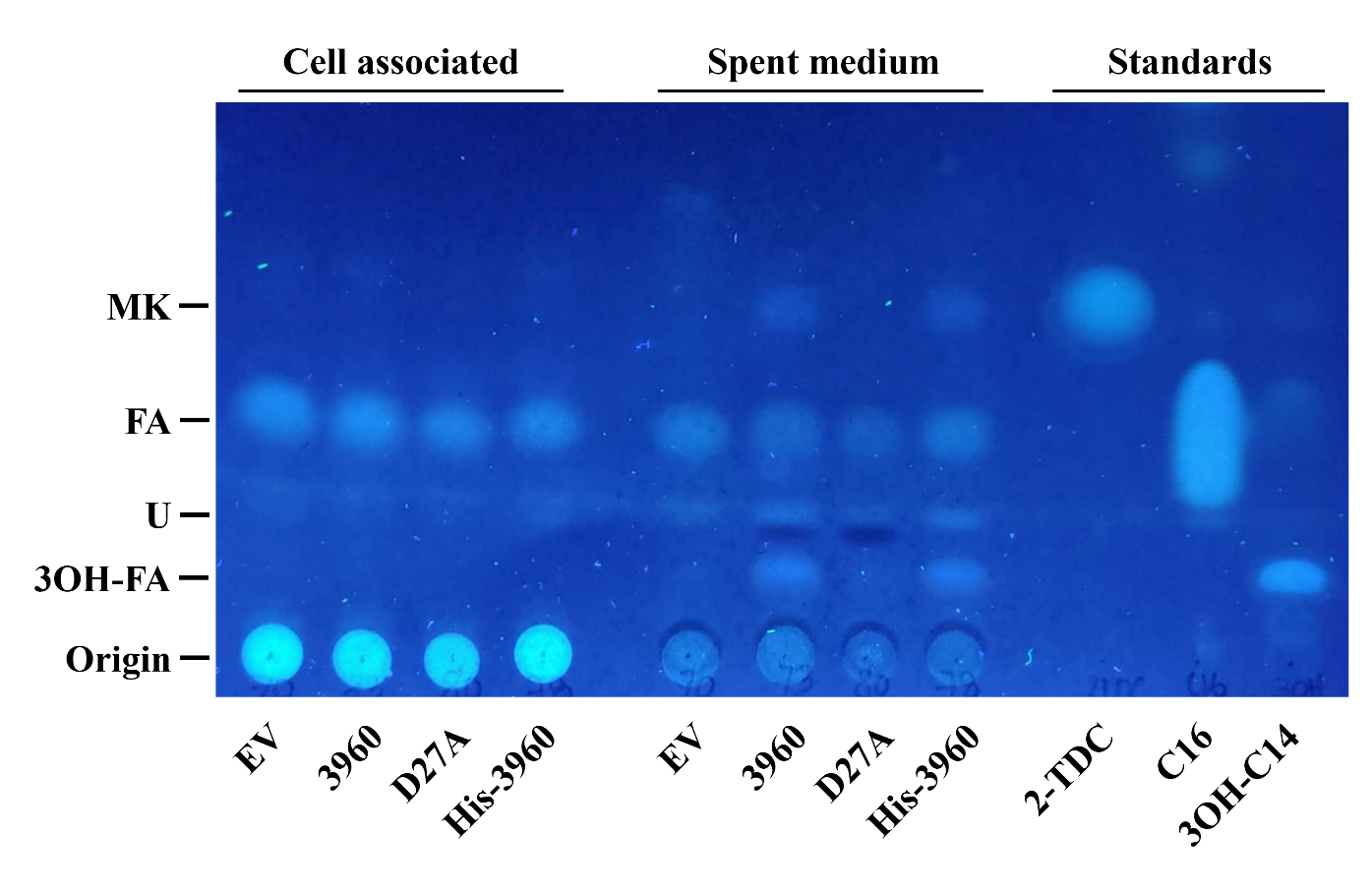
**Supplementary Figure 1**

Primuline staining of TLC separation of lipids extracted from cells (cell associated) or from the spent medium (spent medium) of *E. coli* BL21(DE3) × pLysS cells containing the empty vector pET17b (EV), the SMc03960-expressing construct pIML50 (3960), the construct pGRHD27A which expresses the site directed mutant protein SMc03960_D27A (D27A), or the His-SMc03960-expressing construct pGRH01 (His-3960). As standards, 1 µg of 2-tridecanone (2-TDC), palmitic acid (C16) or 3-hydroxy-myristic acid (3OH-C14) were separated along with lipid extracts. Each strain was grown in 20 ml of M9 minimal medium, IPTG was added at OD = 0.4 and after 4 h, the cell pellet and spent medium were separated by centrifugation. Cell associated lipids were extracted according to the method of Bligh and Dyer [1] and lipids from spent media were extracted with acidified ethyl acetate. Each lane was loaded with the extract corresponding to 6 ml of culture. TLC was developed in hexane/ethyl acetate/acetic acid (70:30:4 [vol/vol/vol]) (same system as for TLC shown in Figure 2A). The separated lipids were visualized under ultraviolet (UV, 336 nm) light after staining with primuline (0.05% in acetone/water [80/20 v/v]). A representative TLC is shown out of 3 repetitions. The origin and migration of products with mobility of methylketones (MK), free fatty acids (FA), and 3-hydroxylated fatty acids (3OH-FA) are indicated. U: unidentified compound.


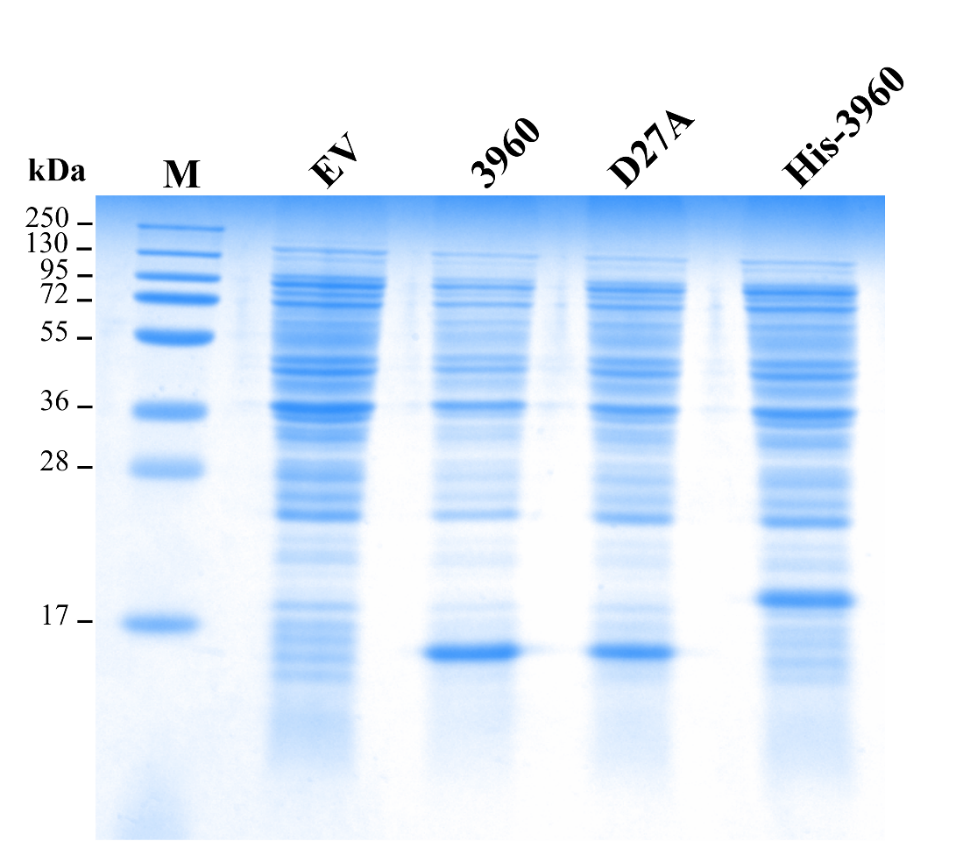


**Supplementary Figure 2****.**

Analysis by 15% SDS-PAGE of total proteins from *E. coli* BL21(DE3) × pLysS cells containing the empty pET17b vector (EV), the SMc03960-expressing construct pIML50 (3960), the construct pGRHD27A which expresses the site-directed mutant protein SMc03960_D27A (D27A), or the His-SMc03960-expressing construct pGRH01 (His-3960). Cultures were grown as described in Supplementary Figure 1. Four hours after induction, 250 µl of cultures were centrifuged and the pellet treated with 25 µl of 1 x SDS-treatment buffer (0.06 M Tris-HCl, 10% glycerol, 2% SDS, and 5% mercaptoethanol at pH 6.8). Aliquots of 4 μl were used for SDS-PAGE performed as described by Laemmli [2]. Each lane contains proteins obtained from 40 µl of culture. The gel was stained with Coomassie Blue.


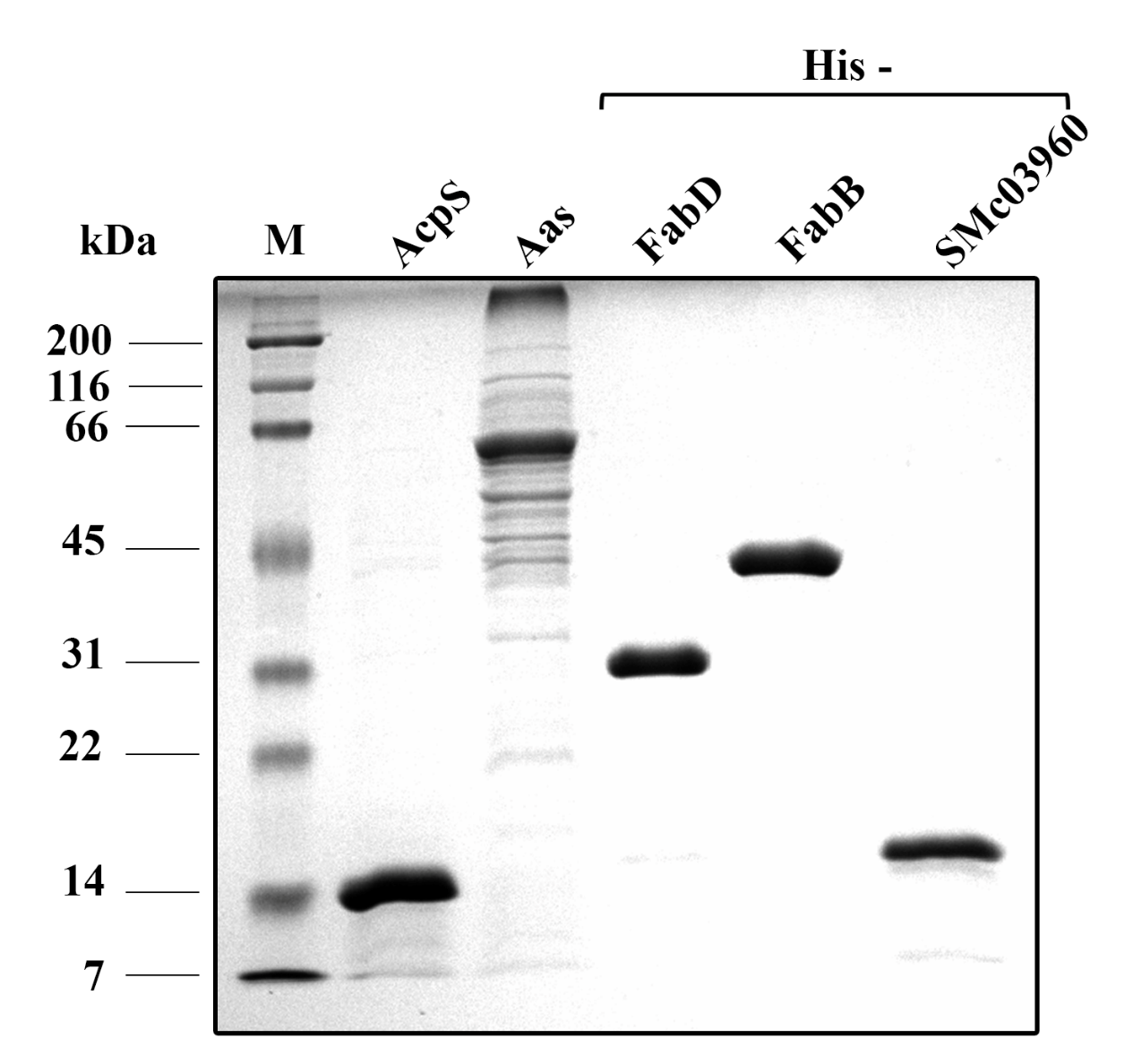


**Supplementary Figure 3**

Analysis by 12% SDS-PAGE of purified proteins and enriched fraction of Aas used in *in vitro* assays. The theoretical molecular weight (MW) of the proteins in kDa are: AcpS: 14.9, Aas: 80.6*, His-FabD: 35, His-FabB: 45.5, and His-SMc03960: 19.2. On the left, MW of the standard proteins is indicated. Samples were mixed with an equal amount of 2x SDS-treatment buffer. SDS-PAGE was performed as described by Laemmli [2] and stained with Coomassie Blue. *Note: Aas protein migrates similarly to the standard of 66 kDa [3].

**Supplementary Table 1.** Effect of *smc03960* overexpression on the relative abundance of methylketones, fatty acids and fatty acid methyl esters (FAMEs) present in spent culture media from *E. coli* BL21(DE3) x pLysS cultures carrying either the empty vector pET17b or the *smc03960*-expressing construct pIML50.^*^

| Compound | | **pET17b** | **pIML50** | **Ratio pIML50/pET17b** |
| --- | --- | --- | --- | --- |
| **Methylketones^a^** | 2-UDC | Nd^b^ | 37.7±24.2 | High effect |
|  | 2-TDC | 1.2±0.1 | 145±31.8 | 120.83 |
|  | 2-PDC | 0.5±0.04 | 74.6±14 | 149.20 |
| **Fatty acids** | C8:0 | 32±2.7 | 34±7.2 | 1.06 |
|  | C9:0 | 39.3±6.8 | 33±3.8 | 0.84 |
|  | C10:0 | 23.3±3.8 | 26.3±2.4 | 1.13 |
|  | C12:0 | 41±2.7 | 59.7±1.5 | 1.46 |
|  | C14:0 | 42±14.6 | 64.3±9.7 | 1.53 |
|  | C15:0 | 4.3±0.9 | 5.3±0.9 | 1.23 |
|  | C16:0 | 147.7±40.2 | 163.7±19.8 | 1.11 |
|  | C18:0 | 91.7±22.8 | 74±17.7 | 0.81 |
|  | C12:1 | 15±2.7 | 69±9.9 | 4.60 |
|  | C14:1 | 27±0.6 | 99.7±9.5 | 3.69 |
|  | C16:1 | 2.7±1.2 | 23.3±1.9 | 8.63 |
|  | C18:1 | 9.7±5.2 | 71±9.8 | 7.34 |
|  | C19:0 cyp^d^ | 25.7±8.4 | 65.7±14 | 2.56 |
|  | 3OH-C14:0 | 13±2.1 | 136±20.2 | 10.46 |
|  | 3OH-C16:0 | 5.3±5.3^c^ | 71.7±8.1 | 13.52 |
|  | **Σ SFA**^e^ | 465.3±77.9 | 733.7±47.4 | 1.57 |
|  | **Σ UFA**^f^ | 54.3±6.7 | 263±26.9 | 4.84 |
| **FAMEs** | C14:0 | 4±2 | 5±2.5 | 1.25 |
|  | C16:0 | 84.7±40.1 | 83.7±38.8 | 0.99 |
|  | C18:1 | 185±92.7 | 176.3±85.3 | 0.94 |
|  | C18:0 | 34.3±6.9 | 72±9 | 2.09 |

^*^Values represent the average and standard error obtained in three independent experiments. Ratio pIML50/pET17b was calculated by dividing the average of the relative abundance obtained for pIML50 by the average relative abundance obtained for the strain carrying the empty vector pET17b. ^a^ Data relative to methylketones are also presented as mg/L in Figure 2B; ^b^ nd, not detected; ^c^ 3OH-C16:0 was detected only in one out of three independent cultures of *E. coli* carrying pET17b; ^d^ cyp, cyclopropane; ^e^ SFA, saturated fatty acids; ^f^ UFA, unsaturated fatty acids.

**Supplementary Table 2.** *S. meliloti* genes coding for proteins with thioesterase domains.

| **Locus in GR4 or Rm1021** | **Description of the encoded protein ^a^**  **(Size in aas)** | **Thioesterase domain/s (interval)** | **Orthologs of known function (% identity) [Reference]** |
| --- | --- | --- | --- |
| *C770_GR4pD0069*  *smb21634* | Acyl-coenzyme A thioesterase (167) | YciA (8-149) | Ec_YciA (29) [4] |
| *C770_GR4Chr0324*  *smc00396* | Conserved hypothetical protein (150) | PaaI_thioesterase (11-146) | Ec_YigI (31) [5] |
| *C770_GR4Chr0884*  *smc00967* | Conserved hypothetical protein (133) | FadM (4-133)  4HBT (9-111) | Ec_YbgC (28) [6]  Ec_FadM (22) [7] |
| *C770_GR4Chr2309*  *smc01638^b^* | carnitine dehydrogenase thioesterase (496) | 4HBT (351-455) | SMc01638^b^ (100) [8] |
| *C770_GR4Chr1258*  *smc01805* | Hypothetical transmembrane protein (143) | PaaI_thioesterase (9-143)  4HBT (54-123) | PA5202 (32) [9] |
| *C770_GR4Chr3273*  *smc03805* | Probable acyl-CoA thioesterase II (294) | TesB (10-291)  Thioesterase_II_repeat2 (26-115) | Ec_TesB (47) [10] |
| *C770_GR4Chr3306*  *smc03836* | Putative arylesterase (229) | TesA (38-217)  PRK10528 (33-203) | Ec_TesA (36) [11] |
| *C770_GR4Chr2869*  *smc03960* | Conserved hypothetical protein (151) | Thio_ybgC (15-139)  FadM (11-149)  4HBT (15-127)  PRK10800 (20-143)  4HBT_2 (24-143) | Ec_YbgC (39) [6]  Sh_MKS2 (23) [12]  Bn_YneP (25) [13] |
| *C770_GR4Chr1952*  *smc04228* | Putative acyl-CoA thioester hydrolase (129) | YciA (7-126)  BFIT_BACH (7-126)  PRK10694 (7-129)  4HBT (26-96) | Ec_YciA (49) [4] |

^a^ The proteins encoded by the corresponding genes in GR4 and Rm1021 are 100% identical; ^b^ *S. meliloti* thioesterase biochemically characterized by Bazire et al. [8].

**Supplementary Table 3.** Bacterial strains and plasmids used in this study.

| **Strain/plasmid** | **Relevant characteristics^a^** | **Reference/source** |
| --- | --- | --- |
| **Strains**  ***E. coli*** |  |  |
| DH5α | *supE44,* Δ*lacU169, f80, lacZ*Δ*M, 5hsdR171, recA1, endA1, gyrA96, thi-1, relA1* | [14] |
| S17-1 | *thi, pro, recA, hsdR, hsdM, RP4-2-Tc::Mu-Km::Tn7* | [15] |
| BL21(DE3) | F^−^*ompT hsdSB*(rB^−^, mB^−^) *gal dcm* (DE3) | [16] |
| ***S. meliloti*** |  |  |
| GR4 | Wild type strain | [17] |
| GFDC | GR4 (∆*fadD*::Km), Km^r^ | M.J. Soto |
| GΔ3960 | GR4 (∆*smc03960*::SmSp), Sm^r^, Sp^r^ | This work |
| GFDΔ3960 | GFDC (∆*smc03960*::SmSp), Km^r^, Sm^r^, Sp^r^ | This work |
| **Plasmids**  pBluescript KS(+) | Cloning vector, Ap^r^ | Stratagene |
| pHP45Ω | Plasmid containing Sm/Sp cassette, Ap^r^, Sm^r^, Sp^r^ | [18] |
| pK18*mobsacB* | Suicide plasmid, Km^r^ | [19] |
| pET9a | Expression vector, Km^r^ | Novagen |
| pET17b | Expression vector, Cb^r^ | Novagen |
| pET16b | Expression vector conferring N-terminal His-tag to expressed proteins, Cb^r^ | Novagen |
| pLysS | Plasmid for production of T7 lysozyme for repression of T7 polymerase, Cm^r^ | [20] |
| pRK404 | Broad host-range vector, Tc^r^ | [21] |
| pNG28 | pET17b cloned in pRK404, Tc^r^, Cb^r^ | [22] |
| pBSΔ3960 | ∆*smc03960* in pBSKS(+), Ap^r^ | This work |
| pBSΔ3960SS | ∆*smc03960*::SmSp in pBSKS(+), Ap^r^ | This work |
| pK18Δ3960SS | ∆*smc03960*::SmSp in pK18*mobsacB*, Km^r^, Sm^r^, Sp^r^ | This work |
| pK18fadDCKm | ∆*fadD*::Km in pK18*mobsacB*, Km^r^ | [23] |
| pTB5035 | *acpP_Sm_* in pET9a, Km^r^ | [24] |
| pAL20 | *acpS_Sm_* in pET9a, Km^r^ | [[25](#_ENREF_23)] |
| pAasH | *aas_Ec_* in pET28a, Km^r^ | [3] |
| pSBD02 | *fabD_Sm_* in pET16b, Cb^r^ | [[25](#_ENREF_23)] |
| pCSB10 | *fabB_Sm_* in pET16b, Cb^r^ | This work |
| pDJ1 | *smc03960* in pET9a, Km^r^ | This work |
| pIML50 | *smc0396*0 in pET17b, Cb^r^ | This work |
| pIML55 | pIML50 into pRK404, Tc^r^, Cb^r^ | This work |
| pGRH01 | *smc03960* in pET16b, Cb^r^ | This work |
| pGRHD27A | site-directed mutant (D27A) of *smc03960* in pET17b, Cb^r^ | This work |

^a^ Km^r^, Sm^r^, Sp^r^, Ap^r^, Cb^r^, Cm^r^, and Tc^r^ indicate kanamycin, streptomycin, spectinomycin, ampicillin, carbenicillin, chloramphenicol, and tetracycline resistance, respectively.

**Supplementary Table 4.** Primers used to obtain a mutant strain and plasmid constructs.

| **Primer name** | **Sequence (5’ to 3’)^a^** | **Use** |
| --- | --- | --- |
| 75 | ATATCTAGATGCGTTTCCGTCATCTGG (*Xba*I) | Deletion of *smc03960* |
| 76 | AAAGGATCCGGCAAGCGAAATCAGTGAC (*Bam*HI) |  |
| 77 | AAAGGATCCTGGCTGCGCAAGCGGC (*Bam*HI) |  |
| 78 | GTGGATTTCGCCGAATTCACG (*Eco*RI) |  |
| 186 | AGGAATACATATGTCACTGATTTCGCTTGCCG (*Nde*I) | Amplification of *smc03960* |
| 187 | AAAGGATCCTCACGCGGAAGCCGCTTGC (*Bam*HI) |  |
| FabB/N | AGGAATACATATGAGACGGGTTGTTGTAACGGGCCTC (*Nde*I) | Amplification of *fabB (smc000327)* |
| FabB/C | AAAGGATCCTTATCCGTTATAGCGCTGGAAGACGAG (*Bam*HI) |  |
| a80c_FW | TATTACGAGGACACCGCTTTTTCCGGCGTCGTC | Site-directed mutagenesis D27A in SMc03960 |
| a80c_R | GACGACGCCGGAAAAAGCGGTGTCCTCGTAATA |  |

^a^ Restriction sites (underlined) are given in parentheses.

**References**

1. Bligh, E.G. and Dyer, W.J. (1959) A rapid method of total lipid extraction and purification. *Can. J. Biochem. Physiol.* **37**, 911-917 doi: 10.1139/o59-099.

2. Laemmli, U.K. (1970) Cleavage of structural proteins during the assembly of the head of bacteriophage T4. *Nature* **227**, 680-685 doi: 10.1038/227680a0.

3. Shanklin, J. (2000) Overexpression and purification of the *Escherichia coli* inner membrane enzyme acyl-acyl carrier protein synthase in an active form. *Protein Expr. Purif.* **18**, 355-360 doi: 10.1006/prep.2000.1206.

4. Zhuang, Z., Song, F., Zhao, H., Li, L., Cao, J., Eisenstein, E. et al. (2008) Divergence of function in the hot dog fold enzyme superfamily: the bacterial thioesterase YciA. *Biochemistry* **47**, 2789-2796 doi: 10.1021/bi702334h.

5. Schmidt, M., Proctor, T., Diao, R. and Freddolino, P.L. (2022) *Escherichia coli* YigI is a conserved Gammaproteobacterial acyl-CoA thioesterase permitting metabolism of unusual fatty acid substrates. *J. Bacteriol.* **204,** e0001422 doi: 10.1128/jb.00014-22.

6. Gully, D. and Bouveret, E. (2006) A protein network for phospholipid synthesis uncovered by a variant of the tandem affinity purification method in *Escherichia coli*. *Proteomics* **6**, 282-293 doi: 10.1002/pmic.200500115.

7. Ren, Y., Aguirre, J., Ntamack, A.G., Chu, C. and Schulz, H. (2004) An alternative pathway of oleate β-oxidation in *Escherichia coli* involving the hydrolysis of a dead end intermediate by a thioesterase. *J. Biol. Chem.* **279**, 11042-11050 doi: 10.1074/jbc.M310032200.

8. Bazire, P., Perchat, N., Darii, E., Lechaplais, C., Salanoubat, M. and Perret, A. (2019) Characterization of L-carnitine metabolism in *Sinorhizobium meliloti*. *J. Bacteriol.* **201**, e00772-18 doi: 10.1128/jb.00772-18.

9. Gonzalez, C.F., Tchigvintsev, A., Brown, G., Flick, R., Evdokimova, E., Xu, X. et al. (2012) Structure and activity of the *Pseudomonas aeruginosa* hotdog-fold thioesterases PA5202 and PA2801. *Biochem. J.* **444**, 445-455 doi: 10.1042/BJ20112032.

10. Naggert, J., Narasimhan, M.L., DeVeaux, L., Cho, H., Randhawa, Z.I., Cronan, J.E. et al. (1991) Cloning, sequencing, and characterization of *Escherichia coli* thioesterase II. *J. Biol. Chem.* **266**, 11044-11050 doi: 10.1016/S0021-9258(18)99125-8.

11. Cho, H. and Cronan, J.E. (1993) *Escherichia coli* thioesterase I, molecular cloning and sequencing of the structural gene and identification as a periplasmic enzyme. *J. Biol. Chem.* **268**, 9238-9245 doi: 10.1016/S0021-9258(18)98341-9.

12. Yu, G., Nguyen, T.T., Guo, Y., Schauvinhold, I., Auldridge, M.E., Bhuiyan, N. et al. (2010) Enzymatic functions of wild tomato methylketone synthases 1 and 2. *Plant Physiol.* **154**, 67-77 doi: 10.1104/pp.110.157073.

13. Zhu, M., Xu, X., Li, Y., Wang, P., Niu, S., Zhang, K. et al. (2019) Biosynthesis of the nematode attractant 2-heptanone and its co-evolution between the pathogenic bacterium *Bacillus nematocida* and non-pathogenic bacterium *Bacillus subtilis*. *Front. Microbiol.* **10**, 1489 doi: 10.3389/fmicb.2019.01489.

14. Hanahan, D. (1983) Studies on transformation of *Escherichia coli* with plasmids. *J. Mol. Biol.* **166**, 557-580 doi: 10.1016/s0022-2836(83)80284-8.

15. Simon, R., Priefer, U. and Pühler, A. (1983) A broad host range mobilization system for *in vivo* genetic-engineering: transposon mutagenesis in Gram-negative bacteria. *Nat. Biotechnol.* **1**, 784-791 doi: 10.1038/nbt1183-784.

16. Studier, F.W., Rosenberg, A.H., Dunn, J.J. and Dubendorff, J.W. (1990) Use of T7 RNA polymerase to direct expression of cloned genes. *Methods Enzymol.* **185**, 60-89 doi: 10.1016/0076-6879(90)85008-c.

17. Casadesús, J. and Olivares, J. (1979) Rough and fine linkage mapping of the *Rhizobium meliloti* chromosome. *Mol. Gen. Genet.* **174**, 203-209 doi: 10.1007/BF00268356.

18. Prentki, P. and Krisch, H.M. (1984) *In vitro* insertional mutagenesis with a selectable DNA fragment. *Gene* **29**, 303-313 doi: 10.1016/0378-1119(84)90059-3.

19. Schäfer, A., Tauch, A., Jager, W., Kalinowski, J., Thierbach, G. and Pühler, A. (1994) Small mobilizable multi-purpose cloning vectors derived from the *Escherichia coli* plasmids pK18 and pK19: selection of defined deletions in the chromosome of *Corynebacterium glutamicum*. *Gene* **145**, 69-73 doi: 10.1016/0378-1119(94)90324-7.

20. Studier, F.W. (1991) Use of bacteriophage T7 lysozyme to improve an inducible T7 expression system. *J. Mol. Biol.* **219**, 37-44 doi: 10.1016/0022-2836(91)90855-z.

21. Scott, H.N., Laible, P.D. and Hanson, D.K. (2003) Sequences of versatile broad-host-range vectors of the RK2 family. *Plasmid* **50**, 74-79 doi: 10.1016/s0147-619x(03)00030-1.

22. González-Silva, N., López-Lara, I.M., Reyes-Lamothe, R., Taylor, A.M., Sumpton, D., Thomas-Oates, J. et al. (2011) The dioxygenase-encoding *olsD* gene from *Burkholderia cenocepacia* causes the hydroxylation of the amide-linked fatty acyl moiety of ornithine-containing membrane lipids. *Biochemistry* **50**, 6396-6408 doi: 10.1021/bi200706v.

23. Nogales, J., Domínguez-Ferreras, A., Amaya-Gómez, C.V., van Dillewijn, P., Cuéllar, V., Sanjuan, J. et al. (2010) Transcriptome profiling of a *Sinorhizobium meliloti fadD* mutant reveals the role of rhizobactin 1021 biosynthesis and regulation genes in the control of swarming. *BMC Genomics* **11**, 157 doi: 10.1186/1471-2164-11-157.

24. López-Lara, I.M. and Geiger, O. (2000) Expression and purification of four different rhizobial acyl carrier proteins. *Microbiology* **146**, 839-849 doi: 10.1099/00221287-146-4-839.

25. Ramos-Vega, A.L., Dávila-Martínez, Y., Sohlenkamp, C., Contreras-Martínez, S., Encarnación, S., Geiger, O. et al. (2009) SMb20651 is another acyl carrier protein from *Sinorhizobium meliloti*. *Microbiology* **155**, 257-267 doi: https://doi.org/10.1099/mic.0.022079-0.
